# Impact of Viral Factors on Subcellular Distribution and RNA Export Activity of HIV-1 Rev in Astrocytes 1321N1

**DOI:** 10.1371/journal.pone.0072905

**Published:** 2013-09-04

**Authors:** Atoshi Banerjee, Ronald Benjamin, Sharmistha Banerjee

**Affiliations:** Department of Biochemistry, School of Life Sciences, University of Hyderabad, Hyderabad, Andhra Pradesh, India; Lady Davis Institute for Medical Research, Canada

## Abstract

CNS associated cells are permissive to HIV-1 infection, but poor in virus production due to attenuated Rev activity. The temporal and the spatial distribution of Rev in human astrocyte 1321N1 and glioblastoma GO-G-CCM were monitored for explaining the reduced Rev activity and low viral production during HIV-1 infection. Rev remained localized to the nuclei of these cells upon infection, attenuating its export activity, as manifested by low copy number of RRE-containing viral-mRNA in the cytoplasm of these cells. In contrast to infection, when Rev alone was transiently expressed, it localized in the cytoplasm of 1321N1. The localization changed to the nucleus when Rev was expressed in the presence of other viral proteins through pro-viral DNA pNL4-3. This study, for the first time, revealed the impact of other HIV-1 proteins apart from host factors in regulating the subcellular localization of Rev in astrocytes and hence the fate of HIV-1 infection in these cells.

## Introduction

Human Immunodeficiency Virus 1 (HIV-1) has a small genome of approximately 9.8 kb size that encodes for fifteen proteins, either by utilizing different Open Reading Frames (ORFs) or by differential splicing. Completely spliced viral mRNA leave the host nucleus and are translated into three early proteins namely Nef, Tat and Rev in the cytoplasm. The unspliced and the partially spliced viral mRNA which encode structural and the accessory proteins, are exported to the cytoplasm with the help of early protein Rev. Rev (Regulator of virion expression) has a nuclear localization signal (NLS) as well as a nuclear export signal (NES). Inside the nucleus, Rev binds to Rev Response Element (RRE) on viral mRNAs and transports them across the nuclear membrane for the expression of other HIV-1 proteins [1], [2], [3]. The Rev deficient virus cannot form new virion particles due to inefficient molecular export of unspliced viral mRNA to the cytoplasm, signifying the role of Rev in the viral life cycle [1]. As Rev shuttles between the nucleus and the cytoplasm for efficient transportation of viral mRNA, delayed expression or altered compartmentalization of Rev can influence the degree of HIV-1 infection in a host cell.

Though HIV-1 is capable of infecting several cell types, the integration of pro-virus, viral replication kinetics, virus particle packaging and the virus production vary in these cells. The cells of hematopoietic origin, primarily lymphocytes, mononuclear cells and dendritic cells are considered to be the natural hosts of HIV-1 [4]. Cells of the Central Nervous System (CNS) can also get infected with HIV-1 leading to neuropathogenesis and dementia [5], [6]. Major target cells within the CNS are macrophages and glial cells [7], [8]. The virus was also detected in astrocytes, the characteristic star shaped glial cells of the CNS, during advanced stages of brain infection [9], [10], [11], [12]. Activated CD4+ T lymphocytes, both during mono- or co-infection, are the primary targets of HIV-1 infection, though HIV-1 remains quiescent within the resting CD4+ T lymphocytes [13], [14]. Compared to T lymphocytes, the infectivity of the mononuclear cells is poor, marked by slower replication of the virus due to various host specific obstructions [15], [16]. Viral replication is relatively reduced in the neural cells with no evident cytopathic effects, despite detectable titers of viral RNA in the infected CNS cells [17], [18].

HIV-1 associates with distinct host cellular factors, in both the cytoplasm and the nucleus at various stages of its life cycle [19], [20], [21], which may result in different replication patterns in diverse cells. The cytoscape drawn by high-throughput proteomics study enlists host proteins involved in cell cycle, translation, nucleo-cytoplasmic transport, chromosomal organization and splicing machinery as possible Rev interacting factors [22], [23], [24], [25]. Host proteins, such as, DDX1, DDX3, CRM1, Sam68 and hRIP, have an impact both on Rev function and distribution in the infected host cells [24], [26], [27], [28], [29]. Rev activity has been shown to be impaired in some astrocyte cell line indicating cell specific block [30], [31], [32], [33]. A group of host proteins with common Rev-interacting domain called Rev Interacting HIV Suppressor Proteins (RISP) are known to repress HIV-1 infection by limiting Rev function of viral mRNA transport in TH4-7-5, an astrocytoma cell line [30]. siRNA mediated knockdown of RISP promoted HIV-1 production in these cells. Transiently expressed HIV-1 Rev was observed to accumulate in the cytoplasm of U138MG astrocytoma cells [32]. However, one should note that transient expression of a protein may not mimic the infected state of a cell. In short, clarity on the temporal and the spatial behaviour of Rev upon infection, instead of transient expression, in cells associated with CNS may help us in further understanding the role of cellular environment in the regulation of HIV-1 infection in the nervous system.

In this paper, we have selected four cell types namely T-lymphocytes SUP-T1, monocyte THP-1, astrocyte 1321N1 and glioblastoma GO-G-CCM for comparative studies on the temporal and spatial expression of Rev. The temporal expression of Rev was similar, but the spatial distribution of Rev varied considerably amongst HIV-1 infected cells. While Rev was observed in both the nucleus and the cytoplasm of SUP-T1 and THP-1 cell lines, it localized to the nucleus of 1321N1 and GO-G-CCM cell lines. Nuclear accumulation of Rev in these cell lines affected the Rev activity which was reflected in the deficient translocation of RRE containing viral mRNA across the nuclear membrane to the cytoplasm. The low proportion of RRE containing viral mRNA in the cytoplasm could be correlated with low virus production by these cells. The study was normalized with pro-viral DNA integrated into the genomes of respective cell lines and splice variants of HIV-1 mRNA were quantified to neutralize the impact of the differences in the infectivity and RNA processing amongst different cell lines. It was also observed that the transiently expressed Rev in 1321N1 distributed to the cytoplasm in contrast to the restriction of Rev to the nucleus of the same cell type during infection. We further showed that the subcellular distribution of Rev in astrocyte 1321N1 changed from the cytoplasm to the nucleus when Rev was transiently expressed with other viral proteins through proviral DNA pNL4-3. It is the nuclear restriction of Rev in these cells which led to the reduced export of the RRE containing viral mRNA to the cytoplasm, making astrocytes inefficient for the virus production. We, for the first time, established the impact of other viral proteins apart from host specific cellular factors in influencing the subcellular distribution of Rev and consequently the fate of HIV-1 infection in astrocytes.

## Results

The following HIV-1 permissive cell-lines [34], [35], [36] were used in the study: SUP-T1 (Human T cell lymphoblastoma); THP-1 (human monocytic cell); 1321N1 (human astrocyte) and GO-G-CCM (human glioblastoma). All the cells could be successfully infected, as evident from the expression of Rev inside the infected cells and viral particles produced after 48 hours of infection.

### Rev Expression was not Delayed in Astrocytes or Glial Cells when Compared to T lymphocytes and Monocytes during HIV-1 Infection

**Figure 1:**
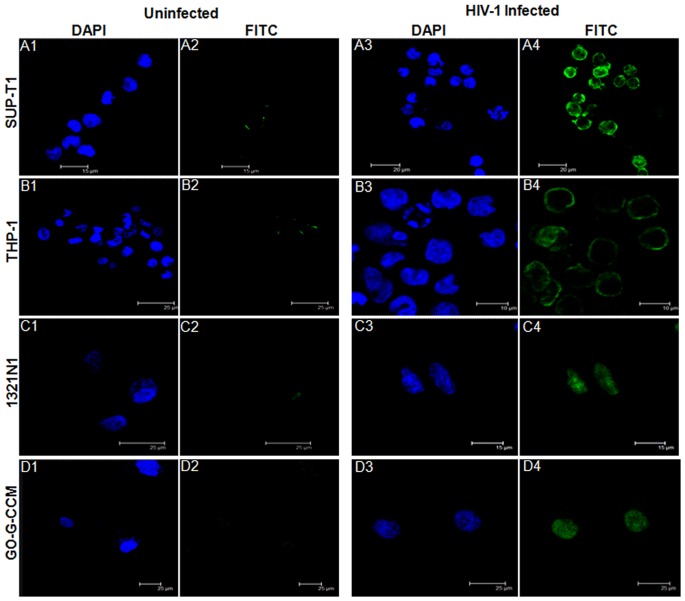
Earliest time point for detection of Rev expression in different cell lines. Cell lines infected with NL4-3 virus were fixed at 1 hour post infection with 3% paraformaldehyde followed by detection with mouse anti HIV-Rev primary antibody, goat anti mouse-FITC (green) secondary antibody and mounted on slides with fluoroshield containing DAPI (blue) for nucleus staining. Uninfected cells were treated similarly and used as control experiments. Panel A: SUP-T1 (A1–A2) uninfected cells; (A3–A4) infected cell line, Panel B: THP-1 (B1–B2) uninfected cells; (B3–B4) infected cell line, Panel C: 1321N1 (C1–C2) uninfected cells; (C3–C4) infected cell line, Panel D: GO-G-CCM (D1–D2) uninfected cells; (D3–D4) infected cell line. DAPI (blue) stains nuclei and FITC (green) shows Rev expression in infected cells. The experiments were done at the least in triplicate and representative pictures are shown here.

The cell lines selected under this study vary with respect to HIV-1 infectivity and viral titers. HIV-1 infection of SUP-T1 produces high viral titers resulting in syncytia formation and cell death [37], whereas THP-1 monocyte/macrophage lineage retains a persistent viral infection and hence serves as a reservoir for HIV-1 latency [38], [39]. Some studies have shown that neural cells, despite their susceptibility to HIV-1 infection, results in poor viral replication with obstructed viral structural gene expression [40], [41], [42], [43]. As it is known that the structural gene expression of HIV-1 depends upon the export activity of Rev, we checked whether Rev expression was prompt or delayed in these cell types. The expression of HIV-1 Rev was monitored till 12 hours post infection. All the cell lines were challenged with high dose (100 ng of p24 equivalent) of HIV-1 (NL4-3) in the presence of polybrene, to compensate for the low viral infection in astrocytes and glial cells [44]. Low abundance of Rev at early hours of infection, made it difficult to be monitored intracellularly [45]. We used immuno-fluorescence technique to detect very low levels of Rev expression using high antibody concentration (1∶50 dilution of primary mouse anti-Rev antibody and 1∶150 dilution for goat anti-mouse FITC conjugated secondary antibody). As a background control, uninfected cells were stained with both primary and secondary antibodies. Background fluorescence (FITC) was not observed under these conditions (Figure 1, Column 2). DAPI was used to stain the nuclei of the cells to indicate the cell integrity. We found that the cells did not lyse with high concentration of the virus and appeared similar to the uninfected cells (Figure 1, Column 1 and 3). Rev expression (FITC) could be detected as early as 1 hour post infection in all the cell lines (Figure 1, Column 4). This suggested that the reported inefficient viral replication in astrocytes or glial cells [40], [41], [42] was not because of the delay in Rev expression. The levels of Rev expression (as evident by the intensity of fluorescence) and the number of cells infected in different cell lines were variable, which may be a reflection of variations in the infectivity of the cells.

### Rev Localized to the Nucleus of HIV-1 Infected Astrocyte and Glial Cell Lines

**Figure 2:**
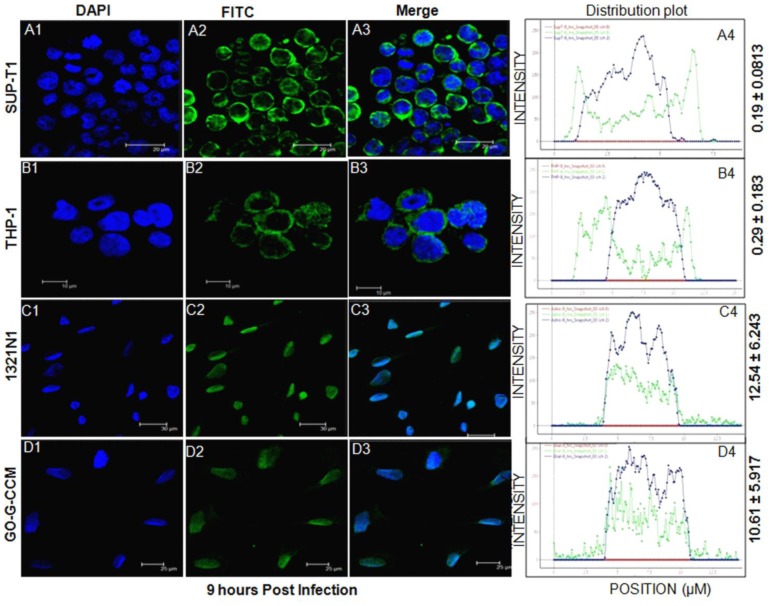
Distribution of Rev 9 hours post infection. Cells were fixed with 3% paraformaldehyde 9 hours post infection followed by detection of Rev with anti-Rev antibody and FITC labeled secondary antibody. DAPI (blue) stains nuclei, FITC (green) stains Rev and merged images of blue and green channels indicates the subcellular localization of Rev protein. The distribution of Rev in a representative cell is plotted using Huygens Essential software twin slicer tool and shown as distribution plot. Ratios of mean intensities of green channel inside and outside the nucleus (blue) were calculated for at least 10–15 cells per field, with minimum of three fields per cell types. The ratios of nucleus to cytoplasmic levels of Rev are given at the end of each panel. Panel A: SUP-T1; Panel B: THP-1; Panel C: 1321N1 and Panel D: GO-G-CCM. The experiments were done at the least in triplicate and representative pictures are shown here.

Having confirmed the expression of Rev, we investigated if the spatial distribution of Rev in these cell lines were different. Rev expression was monitored at various post infection time points in all the cell types. Cells were infected with HIV-1 (NL4-3) virus, fixed with 3% paraformaldehyde at 3, 6 and 9 hours post infection. The nuclei were stained with DAPI (Figure 2, Column 1). The merged images were used to analyze the subcellular localization of Rev (Figure 2, S1 and S2; Column 3). An unbiased graphical analysis of the image was done by using twin slicer tool of Huygens Essential software (explained in Figure S3). Distribution plots depict the distribution of Rev in the cytoplasm and the nuclei of the representative cells for each time point. The Rev distribution was indicated by the green curves (representing FITC staining) in the plots. Nuclear regions in the plots were indicated by the blue curves (representing DAPI staining). Distribution of Rev in the nuclear and the cytoplasmic compartments was evaluated using Leica quantification software as described in materials and methods. The nuclear to cytoplasmic ratio of Rev (N_Rev_/C_Rev_) are indicated beside the plots. Low values of N_Rev_/C_Rev_ points to the pre-dominant cytoplasmic distribution of Rev, while higher values of N_Rev_/C_Rev_ indicated primarily nuclear localization. The image analyses followed by the quantification of Rev distribution, clearly indicated that Rev progressively localized in the cytoplasm of SUP-T1 and THP-1 cells from 3 hours to 9 hours post infection (Figure 2, Panel A and B; Figure S1 and S2, Panel A and B). However, it remained predominantly localized to the nuclei of 1321N1 and GO-G-CCM cells (Figure 2, Panel C and D; Figure S1 and S2, Panel C and D). In order to be sure that Rev remained localized to the nucleus and failed to escape to the cytoplasm over time, we analyzed the distribution of Rev in 1321N1 after 12 hours post infection and found that Rev remained in the nucleus (Figure S4) and was not observed in the cytoplasm of HIV-1 infected 1321N1.

### HIV-1 Infected 1321N1 and GO-G-CCM Cells with Nuclear Restricted Rev had Low RRE Containing Viral mRNA in their Cytoplasm

**Figure 3:**
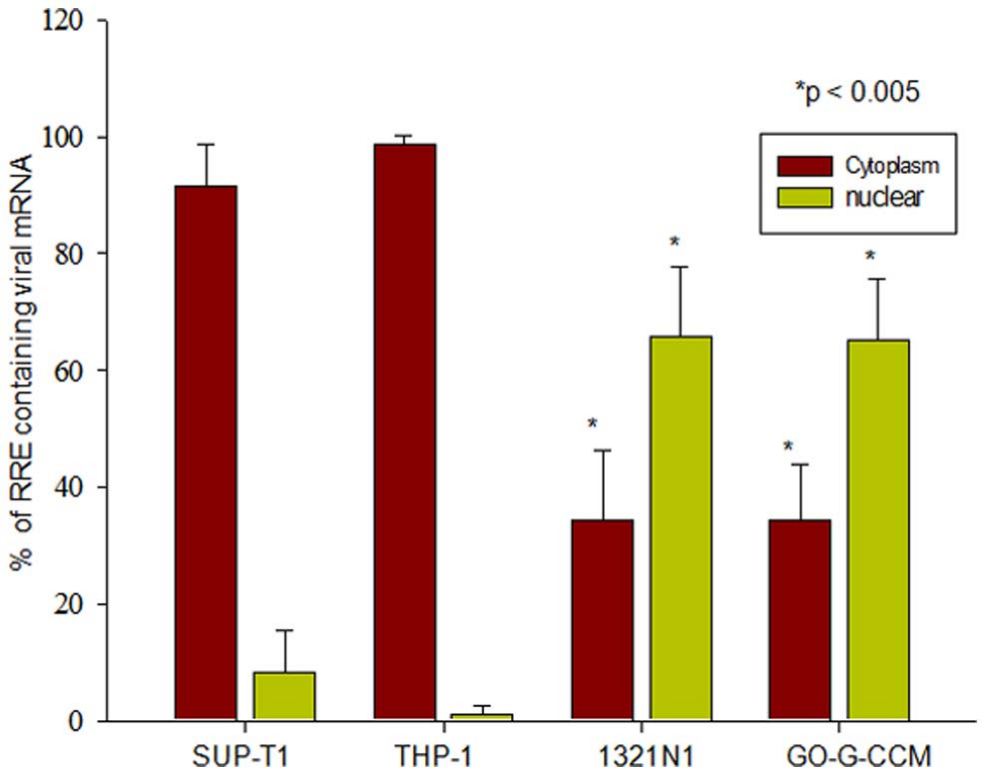
qRT-PCR analysis of RRE-containing viral mRNA in the nuclear and the cytoplasmic fractions of different cell lines. Cells infected with NL4-3 virus were harvested 6 hours post infection. Nuclear and cytoplasmic fractions were separated for RNA extraction. qRT-PCR were performed using RRE specific probes for real time PCR which were FAM labeled. Cytoplasmic and nuclear viral RRE containing mRNA were plotted as percentage of total viral RRE containing mRNA in different cell types Experiments were done in triplicate and ± Standard Deviation (SD) values were determined. The significance is determined by student’s *t* test and p values are denoted.

Rev synthesized upon HIV-1 infection was observed primarily to the nuclei of 1321N1 and GO-G-CCM cells. We further analyzed if any constraints to the movement of Rev across the nuclear membrane affect its RNA transport activity and thereby the virion particle formation. Real time PCR (qRT-PCR) using RRE specific primers was performed for quantifying the RRE containing viral mRNA in the nucleus and the cytoplasm of these cells. The copy numbers of RRE containing viral mRNA distributed in the nuclear and the cytoplasmic fractions were expressed in terms of the percentage of total RRE containing viral mRNA present in a cell. The standard plot used for the quantification is provided in the Figure S5. The copy number of RRE containing viral mRNA in the cytoplasm of SUP-T1 was 91.58±7.12% and 98.75±1.27% in THP-1 cells, indicating that the majority of the viral mRNA was exported to the cytoplasm with negligible RRE containing viral mRNA present in the nucleus of these cells (Figure 3). In contrast, 1321N1 and GO-G-CCM cells had only 34.37±12.04% and 34.23±9.57% respectively of total RRE containing viral mRNA in their cytoplasm (Figure 3). The differences between the distribution of RRE containing viral mRNA in the nucleus and the cytoplasm of 1321N1 and GO-G-CCM cells were statistically significant (p<0.005) (Figure 3). These experiments provided evidence that the nuclear restriction of Rev reduced the export of RRE containing viral mRNA to the cytoplasm of 1321N1 and GO-G-CCM cells.

### Reduced RRE Containing Viral mRNA Export Correlated with Low Virus Production in Astrocyte and Glial Cell Lines upon HIV-1 Infection

**Figure 4:**
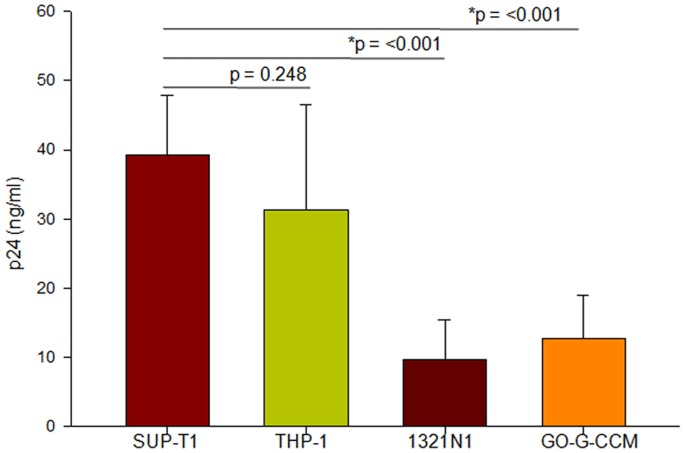
Quantification of p24 equivalents of HIV-1 titers in different cell types. Cells infected with NL4-3 virus were scored for p24 levels from culture supernatant after 48 hours of infection. The viral p24 levels were normalized to the copy number of viral DNA incorporated into the host genome for each of the cell lines following HIV-1 infection. Experiments were done five times and ± SD values were determined. The significance is determined by student’s *t* test and p values are denoted. The bar indicates the groups compared.

As the above experiments confirmed that the nuclear distribution of Rev during HIV-1 infection of 1321N1 and GO-G-CCM cells affected viral RRE containing mRNA export, we further explored, if it would ultimately affect the HIV-1 production by these cells. The viral titers in the culture supernatant were monitored in terms of HIV-1 p24 quantified by ELISA. The p24 levels were normalized with the copy number of viral DNA incorporated into the host genome for each cell type (Figure S6) to neutralize the differences in the viral entry, reverse transcription, and integration. It was observed that the viral p24 levels were drastically reduced in 1321N1 and GO-G-CCM compared to SUP-T1 and THP-1 cells (Figure 4). The highest p24 levels were observed in SUP-T1 (39.31±8.4 ng/ml) followed by THP-1 (31.26±15.18 ng/ml), 1321N1 (9.6±5.8 ng/ml) and GO-G-CCM (12.74±6.2 ng/ml). The relative abundance of the various HIV-1 RNA species (9, 4 and 2 kb) was evaluated to eliminate the possibility of differences in HIV-1 RNA processing amongst the cell lines (Figure S7). No significant differences were observed in the ratio of unspliced (9 kb) or partially (4 kb) spliced RNA to completely spliced (2 kb) RNA amongst all the cell lines.

Put together, these results showed that in SUP-T1 and THP-1 cells, where Rev was evenly distributed in the nucleus and the cytoplasm, the RRE containing viral mRNA could be successfully exported to the cytoplasm for the synthesis of late structural proteins and hence resulted in efficient HIV-1 production by these cells. In 1321N1 and GO-G-CCM cells, the nuclear restriction of Rev resulted in inadequate export of the viral mRNA and hence reduced viral production by these cells. Restriction of Rev to the nucleus, which was not salvaged with time, can be yet another explanation for the compromised HIV-1 propagation in astrocytes and glial cells.

### The Presence of Other Viral Proteins Influenced the Subcellular Distribution of Rev in Astrocyte 1321N1

Rev activity in astrocytes was reported to be constrained in earlier studies [26], [30], [31], [46]. However, there was a huge discordance in the observations and related inferences, either due to different cell lines used or primarily using transient expression of Rev instead of infection by HIV-1. Whenever Rev was expressed transiently in different astrocyte cell lines [31], [32], [33], it remained localized to the cytoplasm, in contrary to our observations where Rev was found mainly distributed to the nucleus upon HIV-1 infection.

**Figure 5:**
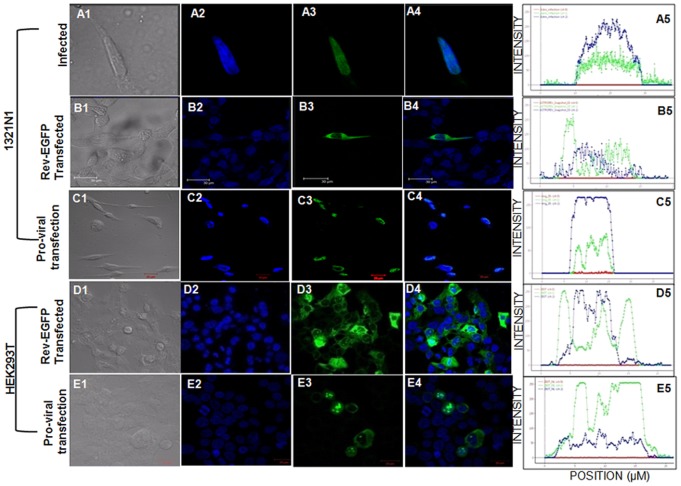
Comparison of Rev distribution in infected and transfected astrocyte 1321N1. Panel A: 1321N1 cells infected with NL4-3 virus; Panel B: 1321N1 cells transfected with Rev-EGFP plasmid; Panel C: 1321N1 cells transfected with pro-viral DNA pNL4-3; Panel D: HEK293T cells transfected with Rev-EGFP plasmid; Panel E: HEK293T cells transfected with pro-viral DNA pNL4-3. DAPI (blue) stains nuclei, FITC (green) stains Rev and Merged images of blue and green channels indicates the subcellular localization of Rev protein. The distribution of Rev in a representative cell is plotted using Huygens Essential software twin slicer tool and shown as distribution plot. All the experiments were done thrice and representative pictures are shown here.

We next asked the question if the presence or absence of other HIV-1 proteins within the same cell type will have an impact on Rev distribution. For this, distribution of transiently expressed Rev through Rev-EGFP vector was compared to the distribution of Rev expressed upon infection in 1321N1 (Figure 5, Panels A and B). It should be noted that the other viral factors like viral proteins, viral RNA etc. are present alongside Rev during HIV-1 infection while Rev, when expressed transiently, is the only viral factor present in a cell. It was clearly seen that Rev expressed upon infection of 1321N1 cells with HIV-1, localized primarily to the nucleus (Figure 5, Panel A; Columns 3, 4 and distribution plot) while, transiently expressed Rev localized predominantly in the cytoplasm (Figure 5, Panel B; Columns 3, 4 and distribution plot). 1321N1 cells were then transfected with pro-viral DNA pNL4-3 to express all the viral proteins including Rev. We observed that in the presence of other viral proteins, localization of Rev changed to the nucleus of 1321N1 as observed during infection (Figure 5, Panel C; Columns 3, 4 and distribution plot). To rule out that the impact of viral proteins on subcellular distribution of Rev is not a cell specific event, HEK293T cells were used as an alternate host. HEK293T cells were transfected with Rev-EGFP or pNL4-3 and the distribution of Rev was monitored (Figure 5, Panel D and E). Empty EGFP vector was used as a control in both the cell lines (Figure S8). It was observed that Rev, whenever expressed accompanied by other viral proteins through pNL4-3 plasmid, lost preference for cytoplasmic distribution and was observed in the nuclei of the majority of transfected HEK293T cells (Figure 5, distribution plots).

Together with the above results, we concluded that the nuclear restriction of Rev influenced by other viral proteins resulted into low RRE containing viral mRNA in the cytoplasm of infected 1321N1 cells leading to reduced HIV-1 production. Although the influence of host factors on the distribution of Rev had been shown previously [26], [46], [47], our work highlights for the first time, the importance of viral proteins in Rev distribution in astrocytes.

## Discussion

In this study we have focused on the time dependent expression and distribution of Rev to understand the reasons behind its reduced activity in astrocytes and glial cells as compared to T lymphocytes and monocytes. From our observations, we concluded that the presence of viral proteins influence the subcellular localization of Rev in astrocytes either directly or indirectly by modulating the host environment, thereby accounts for inefficient export of RRE containing viral mRNA and hence lower virion particle formation by these cells.

Rev is not known to be carried by HIV-1 virion particles and requires to be synthesized early in the life cycle of HIV-1 from completely spliced viral mRNA [48]. The temporal differences in expression of Rev was determined in SUP-T1, THP-1, 1321N1 and GO-G-CCM cell lines, in order to score for the time lag in mRNA transport activity of Rev in these cells. Unlike, other proteins of HIV-1, the information on the time point at which Rev expression commenced was not evident in earlier studies [45]. We observed that the commencement time for Rev expression was about the same in all the cell types (Figure 1). It should be noted that even at the earliest time point of 1 hour post infection, the distribution of Rev was predominantly in the nucleus in 1321N1 and GO-G-CCM (Figure 1). This is possible only when Rev is synthesized in the cytoplasm prior to its nuclear import, suggesting that Rev expression probably begins earlier than 1 hour post infection. We, however, could not capture Rev expression earlier than 1 hour post infection because of insufficient expression of Rev for effective detection in any of the cell lines.

In the later time points of 6 hours and 9 hours post infection, Rev predominantly localized in the cytoplasm of SUP-T1 and THP-1 indicating its free movement across the nuclear membrane, while, it remained primarily localized to the nucleus in 1321N1 and GO-G-CCM (Figure 2, S1 and S2). Understanding that the nuclear localization of Rev should also affect viral mRNA export in astrocytes and glial cells, we compared the levels of RRE containing viral mRNA, in the cytoplasm versus nucleus in all the cell types through qRT-PCR (Figure 3). We observed that only 34% of RRE containing viral mRNA could be detected in the cytoplasm of 1321N1 and GO-G-CCM (Figure 3). Accordingly, the viral titers by 1321N1 and GO-G-CCM cells were also less (Figure 4).

Our experiments showing dissimilar distribution of Rev in SUP-T1, THP-1, 1321N1 and GO-G-CCM provided evidence for the decisive role of host specific cellular factors/environment in influencing Rev distribution, function and consequent production of virion particles. The RRE containing mRNA distribution pattern showed that the Rev activity was reduced in astrocytes and glial cells as compared to T cells and monocytes. The importance of the host factors in influencing Rev activity began with earlier observations, when the defect in the Rev export activity in murine cells could be rescued by fusing uninfected human cells with murine cells [49]. Since then, HIV-1 virus-host interactome has been elucidated both computationally and experimentally [50], [51], [52], [53]. The presence of some of the known Rev co-factors such as DDX1, DDX3, Sam68, hRIP, and CRM1 [24], [26], [27], [28], [29], [54] were checked in all the cell types (Figure S9). We observed that these factors were present at the transcript levels in all the cell types though their levels varied.

During infection, Rev was localized in the nuclei of 1321N1 cells. This was different from some of the reports where Rev localization in astrocyte cell lines like U87MG, U138MG and U373MG was shown in the cytoplasm [32]. These studies used Rev expressed transiently through a vector in the absence of any other viral factors. It further intrigued us to explore the role of viral factors alongside host factors in affecting Rev distribution in astrocytes and hence its impact on viral mRNA export to the cytoplasm. As most of the earlier Rev localization studies were performed using transient expression of Rev through eukaryotic expression vectors, we repeated the study by comparing the distribution of Rev in the same host environment of 1321N1 under three conditions, upon transiently expressing Rev alone, transiently expressing Rev with other viral proteins through pro-viral DNA pNL4-3 and expressing Rev upon HIV-1 infection (Figure 5). It was evident from these experiments that Rev when expressed alone localized in the cytoplasm, but whenever is expressed along with other viral proteins, either through pNL4-3 plasmid or through virus infection, localized primarily to the nucleus (Figure 5, Panel A–C). To rule out that this impact of other viral proteins on Rev distribution was astrocyte specific, as an alternate cellular environment, HEK293T cells were used. HEK293T cells were transfected with Rev-EGFP or pNL4-3 (Figure 5, Panel D and E). We observed, similar to 1321N1 cells, the Rev distribution pattern shifted primarily to the nuclei of HEK293T cells in most of the transfected cells. The experiments involving HEK293T cells re-emphasized the impact of viral proteins on subcellular distribution of Rev. It would be interesting to further explore if the impact of viral proteins on subcellular distribution of Rev is direct or indirect by modulating the host environment.

In conclusion, poor propagation of HIV-1 virus in 1321N1 and GO-G-CCM cells are the consequence of the nuclear localization of Rev which along with host environment is equally influenced by the viral factors. Identification of such host-pathogen interactions at the protein level can improve our understanding of HIV-1 associated neuropathogenesis and can be further explored for therapeutic interventions.

## Materials and Methods

### Ethics Statement

Sources of the cell lines used: HEK293T- a human embryonic kidney cell (Karyala *et al* PLoS One 5: e14408) - gifted by ILS, Hyderabad, India. 1321N1 - Human astrocytoma cell line (Haedicke *et al* PNAS 106: 14040–14045) and GO-G-CCM - Human glioblastoma cells (Dash *et al* Retrovirology 5: 25) - gifted by Prof. A. Kondapi, Dept. of Biotechnology, University of Hyderabad, India. SUP-T1- human T cell lymphoblastic lymphoma (Santos *et al* Retrovirology 9: 65) - gifted by Dr. S. Jameel, ICGEB, Delhi, India. THP-1 human acute monocytic leukemia cell lines (Santos *et al* Retrovirology 9: 65) - from NCCS, Pune, India.

### Cell Culture

Cells used in the study HEK293T, a human embryonic kidney cell [55]; 1321N1, human astrocytoma cell line [35] and GO-G-CCM, human glioblastoma cells [36] were maintained in DMEM media (Invitrogen). SUP-T1, human T cell lymphoblastic lymphoma and THP-1, human acute monocytic leukemia cell lines were maintained in RPMI-1640 media (Invitrogen) [34]. The cells were supplemented with 10% fetal bovine serum (GIBCO, USA) and 1% antibiotic penicillin streptomycin solution (Himedia Laboratories Pvt Ltd, India).

### HIV-1 Virus Production and Infection

HIV-1 virus particles were produced by transient transfection of proviral plasmid pNL4-3 into HEK293T cells using the calcium phosphate method as described earlier [56]. Viral supernatant was collected, filtered through 0.45 µM filter (Millipore), precipitated using PEG 10000 (Sigma Aldrich) and quantified by HIV-1 p24 ELISA kit (ABL, USA) according to the manufacturer’s protocol. For the viral infection, adherent cells were seeded 24 hours prior to infection. All the cell types were infected with NL4-3 HIV-1 virus, with 100 ng of p24 equivalent, in the presence of 8 µg/ml polybrene (Sigma Aldrich, USA) without FBS and antibiotic and kept for 2 hours at 37°C with 5% CO_2_. After 2 hours of infection, cells were washed three times thoroughly with phosphate buffer saline (PBS) to remove free virion particles. Complete media was added to the cells and kept at 37°C with 5% CO_2_.

### Immunocytochemistry

Adherent cells were seeded on coverslips 24 hours prior to the experiment. Suspension cells were treated similar to adherent cells except they were incubated in 1.5 ml tubes. Cells were infected for 2 hours as mentioned earlier. Post infection, the cells were harvested, washed twice with PBS at their respective time points, fixed in 3% paraformaldehyde (Himedia Laboratories Pvt Ltd, India) and kept at 4°C until all the samples were prepared. Fixed cells were washed thrice to remove excess paraformaldehyde and permeabilized by adding ice-cold methanol (Himedia Laboratories Pvt Ltd, India) for 5 min at −20°C. Cells were washed again with PBS and blocked for 1 hour with PBS containing 3% Bovine Serum Albumin (BSA, Sigma Aldrich, USA) at room temperature (RT). Subsequently, cells were incubated with primary antibody monoclonal mouse anti-Rev, at 1∶50 dilution (Santa Cruz Biotechnology, Santa Cruz, CA, USA) in PBS supplemented with 2% BSA for 1 hour. After gentle washing, cells were incubated with secondary antibody goat-anti mouse IgG-FITC conjugated, at 1∶150 dilution (Santa Cruz Biotechnology, Santa Cruz, CA, USA) in the dark for 1 hour at RT. Finally, the cells were washed carefully with PBS, mounted with fluoroshield (Sigma Aldrich, USA) containing DAPI (4, 6-diamidino-2-phenylindole) onto the slides and were viewed under the confocal microscope. As a control, cells without infection were simultaneously treated similarly to eliminate the background staining.

### Measurement of Rev Fluorescence Intensities

To measure the fluorescence intensities, region of interest (ROI) was created in the nucleus and the cytoplasm of almost equal size using Leica quantification software and mean intensities, blue for DAPI and green for FITC conjugated antibody were measured. Three fields were scanned for every time point with each single field consisting of 10 to 15 cells. The ratios were determined by dividing the mean intensities of Rev fluorescence in the nucleus and the cytoplasm. All the experiments were done in triplicate. For further representation, Huygens Essential software was used to plot graph representing the nuclear to cytoplasmic fluorescence intensities of both the blue and the green channel using twin slicer tool.

### qRT-PCR

To measure the HIV-1 unspliced mRNA, infected cells were fractionated into the cytoplasmic and the nuclear fractions and RNA were isolated from each of the fraction using Paris kit (Invitrogen) according to manufactures’ protocol. From both the nuclear and the cytoplasmic fraction, 500 ng of RNA was converted to cDNA. cDNA was prepared using superscript III cDNA synthesis kit (Invitrogen) at 50°C for 1 hour followed by inactivation at 70°C for 15 min. To check for the nuclear and cytoplasmic contamination, we performed semi quantitative RT-PCR for β-actin and pre-GAPDH. Region specific to RRE was chosen to amplify unspliced RNA as it is absent in the spliced RNA. FAM labeled Taqman probe (Invitrogen) was designed against RRE. The RRE template was quantified, to give known copies of RRE product and a standard curve was made based on the Ct values (Figure S5A and B). From the standard curve, RRE copy number of unknown samples were determined and plotted as percentage of the RRE present within the nucleus and the cytoplasm.

### Quantification of Pro-viral Copy Number in the Host Genome

The viral DNA incorporation into the host genome was checked by performing a two step PCR, first using Alu-FP/Gag-RP primer and second using LTR-forward/LTR-Reverse primer sets (Table S1) according to previously described protocol [57] with some modifications.

#### Pre-amplification step

Genomic DNA was isolated from infected cells by Phenol:Chloroform:Isoamylalcohol (25∶24:1) method 6 hours post infection. Hot start PCR was performed with 100 nM Alu-FP, 600 nM Gag-RP and 100 ng of genomic DNA in EmeraldAmp Max HS master mix (Takara) reaction. The PCR was carried out with initial denaturation step at 98°C for 2 minutes followed by 20 cycles of denaturation at 98°C for 30 seconds, annealing at 50°C for 45 seconds and extension at 68°C for 2 minutes.

#### Second step qRT-PCR

HIV-1 integration was quantified by a second round qRT- PCR using 1 µl of the product from the pre-amplification step. qRT-PCR was carried out using LTR-Forward and LTR-Reverse primers (Table S1) in SYBR-Premix Ex Taq (Takara). The PCR conditions used were, initial denaturation step at 95°C for 2 minutes followed by 40 cycles of denaturation at 95°C for 15 seconds, annealing at 55°C for 15 seconds and extension at 60°C for 30 seconds. The DNA region corresponding to the promoter of CXCR4 was used as an internal control to normalize total genomic DNA.

### Transfection

HIV-1 Rev was cloned in EGFP-C3 vector (Clontech) between *Bgl*II and *Sal*I (Fermentas) sites using primers Rev-GFP FP and Rev-GFP RP (Table S1). The clone was confirmed by double digestion followed by sequencing (Eurofins MWG Operon). Rev-GFP vector was transfected into 1321N1 and HEK293T cells (used as a control) using lipofectamine LTX and Plus reagent (Invitrogen) following the manufacturer’s protocol. Media was changed after 4 hours and cells were rested for 36 hours. The expression of GFP tagged Rev was checked using confocal microscope.

### Statistical Analysis

Statistical significance between individual experiments was determined by student’s *t-*test and between the groups was determined by one-way ANOVA. p values <0.05 were considered to be statistically significant. The variance between the groups was determined by one way ANOVA.

## Supporting Information

Figure S1 **Distribution of Rev 3 hours post infection.** Cells were fixed with 3% paraformaldehyde 3 hours post infection followed by detection of Rev with anti-Rev antibody and FITC labeled secondary antibody. DAPI (blue) stains nuclei, FITC (green) stains Rev and merged images of blue and green channels indicate the subcellular localization of Rev protein. The distribution of Rev in a representative cell is plotted using Huygens Essential software twin slicer tool and shown as distribution plot. Ratios of mean intensities of green channel inside and outside the nucleus (blue) were calculated for at least 10–15 cells per field, with minimum of three fields per cell types. The ratios of nucleus to cytoplasmic levels of Rev are given at the end of each panel. Panel A: SUP-T1; Panel B: THP-1; Panel C: 1321N1 and Panel D: GO-G-CCM. The experiments were done at the least in triplicate and representative pictures are shown here.
(TIF)

Figure S2 **Distribution of Rev 6 hours post infection.** Cells were fixed with 3% paraformaldehyde 6 hours post infection followed by detection of Rev with anti-Rev antibody and FITC labeled secondary antibody. DAPI (blue) stains nuclei, FITC (green) stains Rev and merged images of blue and green channels indicate the subcellular localization of Rev protein. The distribution of Rev in a representative cell is plotted using Huygens Essential software twin slicer tool and shown as distribution plot. Ratios of mean intensities of green channel inside and outside the nucleus (blue) were calculated for at least 10–15 cells per field, with minimum of three fields per cell types. The ratios of nucleus to cytoplasmic levels of Rev are given at the end of each panel. Panel A: SUP-T1; Panel B: THP-1; Panel C: 1321N1 and Panel D: GO-G-CCM. The experiments were done at the least in triplicate and representative pictures are shown here.
(TIF)

Figure S3 **Quantification of distribution of Rev in the nuclear and cytoplasmic regions.** A) Confocal image of THP-1 cells expressing Rev upon infection. B) Graphical representation of Rev distribution across the nuclear and cytoplasmic region in a representative THP-1 cell infected with HIV-1. The blue line in the distribution plot pointed to the boundaries of the nucleus and the zone outside the blue line represents cytoplasm. The region covered by the green line indicates Rev expression. If it overlaps with the blue line it indicated fraction of Rev present within the nucleus. The green line on either side of blue line indicated the cytoplasmic fraction of Rev.
(TIF)

Figure S4 **Distribution of Rev 12 hours post infection in astrocyte 1321N1.** Cells were fixed with 3% paraformaldehyde 12 hour post infection followed by detection of Rev with anti-Rev antibody and FITC labeled secondary antibody. DAPI (blue) stains nuclei, FITC (green) stains Rev and merged images of blue and green channels indicate the subcellular localization of Rev protein. The distribution of Rev in a representative cell is plotted using Huygens Essential software twin slicer tool and shown as distribution plot.
(TIF)

Figure S5 **Standard plot for qRT-PCR analysis of RRE-containing viral mRNA in the nuclear and the cytoplasmic fractions.** (A) Fluorescence intensities of known concentrations of vector containing RRE region of viral RNA were plotted against cycle number and threshold was determined by qRT-PCR. (B) Ct values v/s copies of RRE mRNA were plotted and standard graph was made. Copy numbers of RRE in the unknown sample were calculated by plotting the Ct values on the graph.
(TIF)

Figure S6 **Quantification of viral DNA integrated into the host genome.** Histogram representing copy number of viral DNA integrated into the host genome of SUP-T1, THP-1, 1321N1 and GO-G-CCM cells. The DNA region corresponding to the promoter of CXCR4 was used as an internal control to normalize the total genomic DNA. Experiments were done five times and ± SD values were determined. The significance is determined by student’s *t* test and p values are denoted. The bar indicates the groups compared.
(TIF)

Figure S7 **The ratio of unspliced (9 kb) or partially spliced (4 kb) to completely spliced (2 kb) RNA transcript in SUP-T1, THP-1, 1321N1 and GO-G-CCM cells.** Briefly, RNA was extracted from each cell type after 6 hour post infection, qRT-PCR was performed for primers specific to 9 kb, 4 kb and 2 kb HIV-1 RNA species. Ratio of partially (4 kb) spliced or unspliced (9 kb) HIV-1 RNA to completely spliced (2 kb) RNA for respective cell lines were plotted. The ratio of unspliced (9 kb) or (4 kb) spliced HIV-1 RNA to completely spliced (2 kb) RNA in all the cell lines did not differ significantly, with all showing less than 0.5 fold differences. All experiments were done more than three times and error bars represent mean ± SD.
(TIF)

Figure S8 **Control EGFP vector expression in astrocyte 1321N1 and HEK293T cells.** Astrocyte 1321N1 and HEK293T cells were transfected with EGFP vector alone and expression was checked after 48 hours. All the experiments were done thrice and representative pictures are shown here.
(TIF)

Figure S9 **Expression profiles at the transcript levels of known Rev interacting partners.** Semi-quantitative RT-PCR analyses of 6 different genes namely *Sam68, DDX3, DDX1, CRM1, hRIP* and *β-actin* in SUP-T1, THP-1, 1321N1 and GO-G-CCM. β-actin was used as a loading control. All experiments were done three times and a representative gel is shown.
(TIF)

Table S1 **List of primers used in the study.**
(DOCX)

Materials and Methods S1 (DOCX)
