# Supplementary figures and images for: Impact of Viral Factors on Subcellular Distribution and RNA Export Activity of HIV-1 Rev in Astrocytes 1321N1
Source: PLoS One. 2013 Sep 4;8(9):e72905. doi: 10.1371/journal.pone.0072905 (PMC3762830; doi:10.1371/journal.pone.0072905)

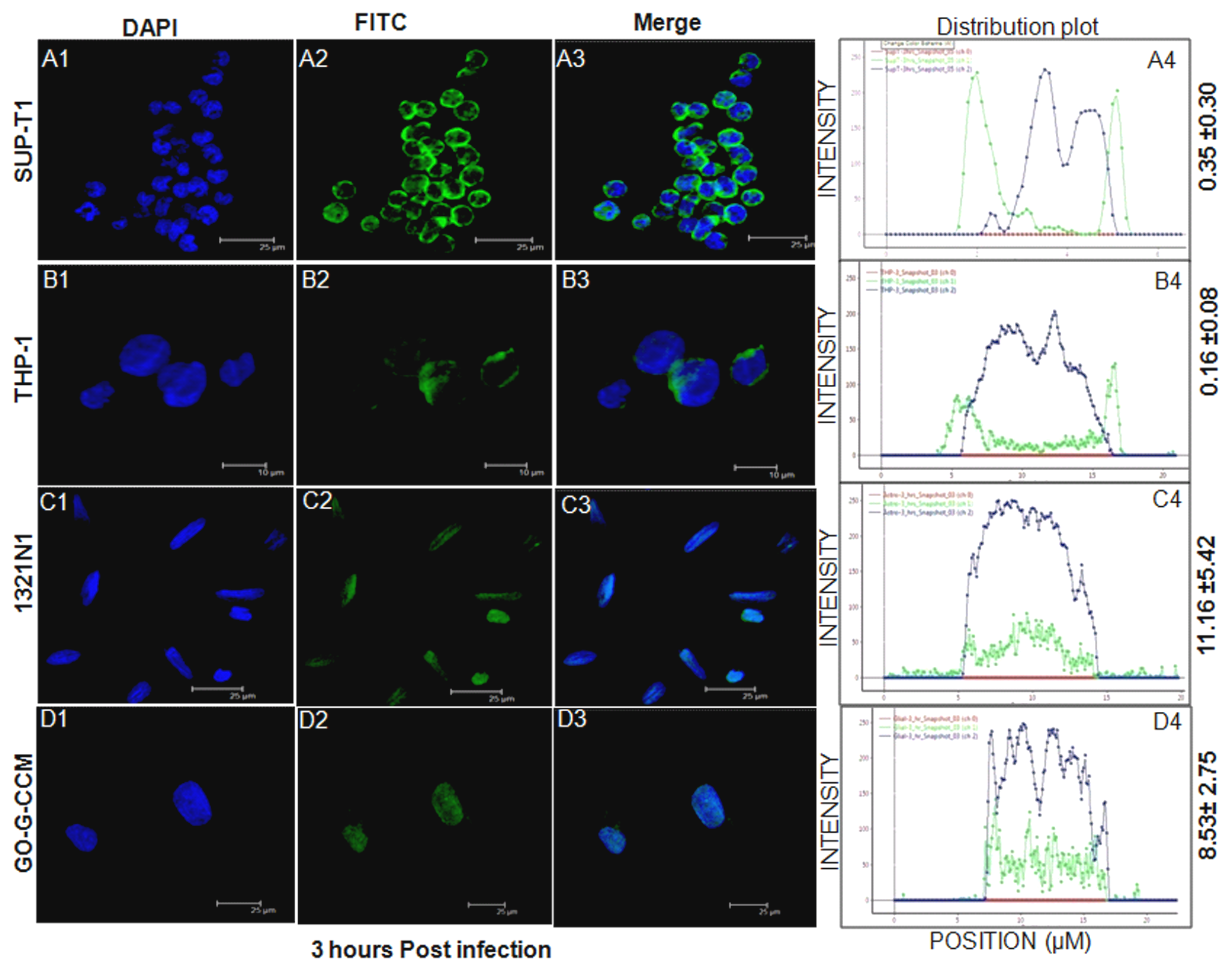

Supplement: Figure S1 — Distribution of Rev 3 hours post infection. Cells were fixed with 3% paraformaldehyde 3 hours post infection followed by detection of Rev with anti-Rev antibody and FITC labeled secondary antibody. DAPI (blue) stains nuclei, FITC (green) stains Rev and merged images of blue and green channels indicate the subcellular localization of Rev protein. The distribution of Rev in a representative cell is plotted using Huygens Essential software twin slicer tool and shown as distribution plot. Ratios of mean intensities of green channel inside and outside the nucleus (blue) were calculated for at least 10–15 cells per field, with minimum of three fields per cell types. The ratios of nucleus to cytoplasmic levels of Rev are given at the end of each panel. Panel A: SUP-T1; Panel B: THP-1; Panel C: 1321N1 and Panel D: GO-G-CCM. The experiments were done at the least in triplicate and representative pictures are shown here. (TIF) [file pone.0072905.s001.tif]

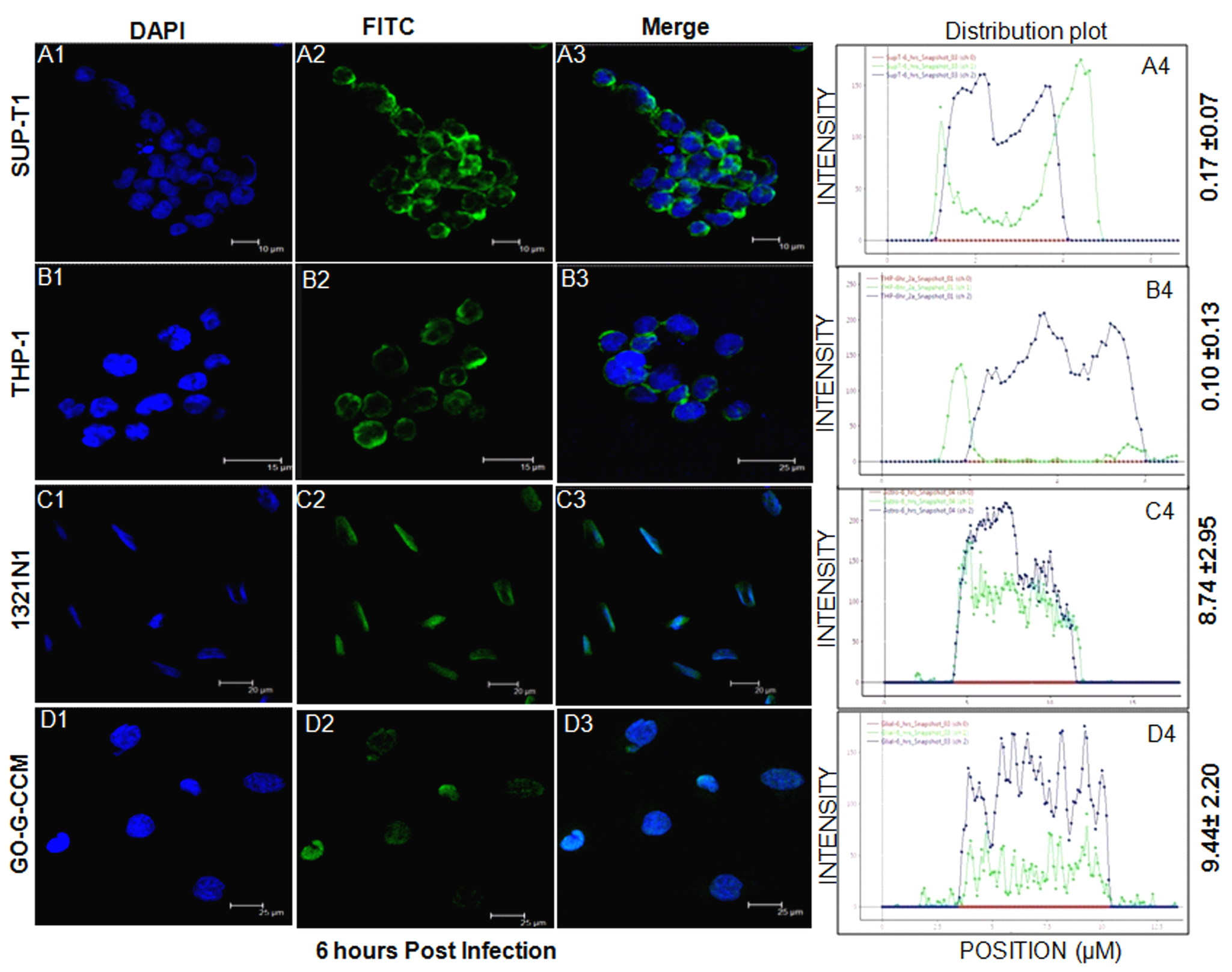

Supplement: Figure S2 — Distribution of Rev 6 hours post infection. Cells were fixed with 3% paraformaldehyde 6 hours post infection followed by detection of Rev with anti-Rev antibody and FITC labeled secondary antibody. DAPI (blue) stains nuclei, FITC (green) stains Rev and merged images of blue and green channels indicate the subcellular localization of Rev protein. The distribution of Rev in a representative cell is plotted using Huygens Essential software twin slicer tool and shown as distribution plot. Ratios of mean intensities of green channel inside and outside the nucleus (blue) were calculated for at least 10–15 cells per field, with minimum of three fields per cell types. The ratios of nucleus to cytoplasmic levels of Rev are given at the end of each panel. Panel A: SUP-T1; Panel B: THP-1; Panel C: 1321N1 and Panel D: GO-G-CCM. The experiments were done at the least in triplicate and representative pictures are shown here. (TIF) [file pone.0072905.s002.tif]

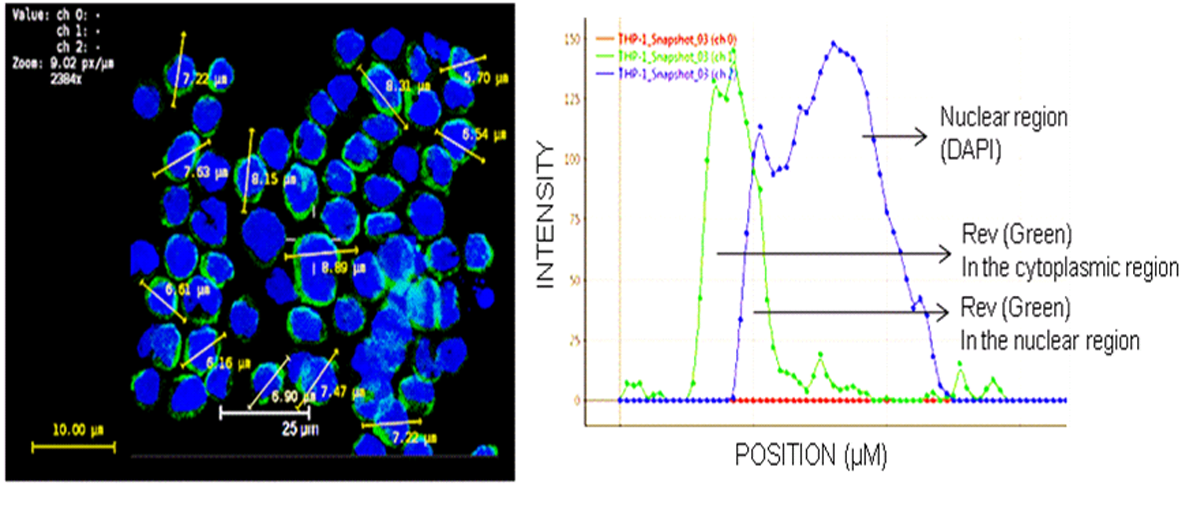

Supplement: Figure S3 — Quantification of distribution of Rev in the nuclear and cytoplasmic regions. A) Confocal image of THP-1 cells expressing Rev upon infection. B) Graphical representation of Rev distribution across the nuclear and cytoplasmic region in a representative THP-1 cell infected with HIV-1. The blue line in the distribution plot pointed to the boundaries of the nucleus and the zone outside the blue line represents cytoplasm. The region covered by the green line indicates Rev expression. If it overlaps with the blue line it indicated fraction of Rev present within the nucleus. The green line on either side of blue line indicated the cytoplasmic fraction of Rev. (TIF) [file pone.0072905.s003.tif]

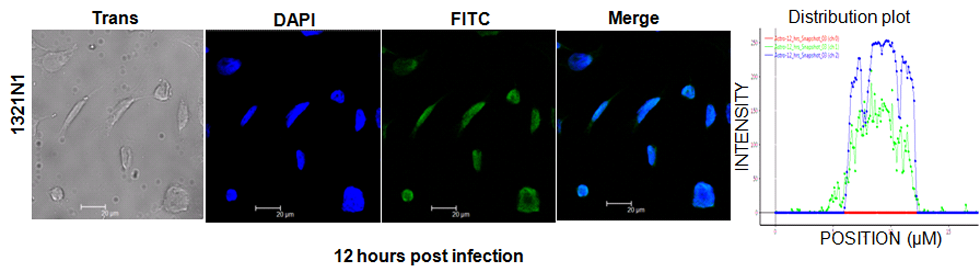

Supplement: Figure S4 — Distribution of Rev 12 hours post infection in astrocyte 1321N1. Cells were fixed with 3% paraformaldehyde 12 hour post infection followed by detection of Rev with anti-Rev antibody and FITC labeled secondary antibody. DAPI (blue) stains nuclei, FITC (green) stains Rev and merged images of blue and green channels indicate the subcellular localization of Rev protein. The distribution of Rev in a representative cell is plotted using Huygens Essential software twin slicer tool and shown as distribution plot. (TIF) [file pone.0072905.s004.tif]

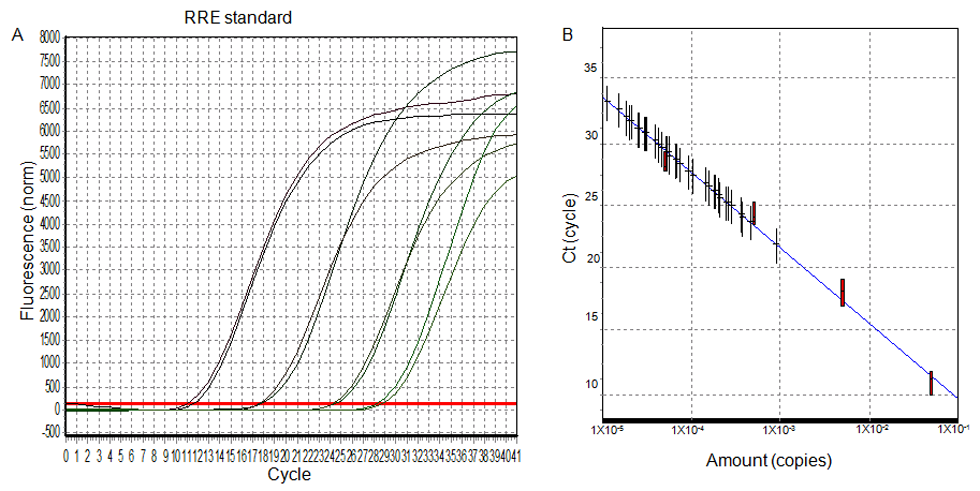

Supplement: Figure S5 — Standard plot for qRT-PCR analysis of RRE-containing viral mRNA in the nuclear and the cytoplasmic fractions. (A) Fluorescence intensities of known concentrations of vector containing RRE region of viral RNA were plotted against cycle number and threshold was determined by qRT-PCR. (B) Ct values v/s copies of RRE mRNA were plotted and standard graph was made. Copy numbers of RRE in the unknown sample were calculated by plotting the Ct values on the graph. (TIF) [file pone.0072905.s005.tif]

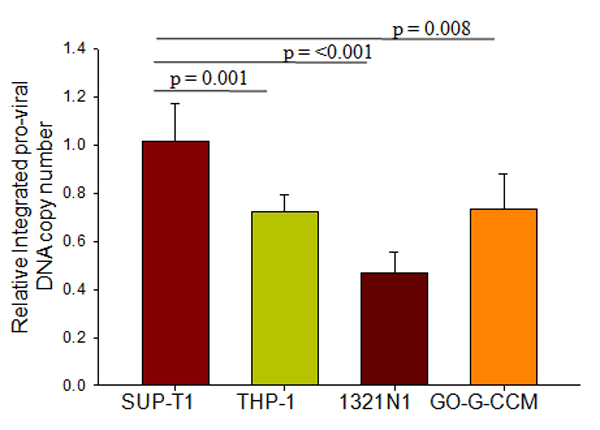

Supplement: Figure S6 — Quantification of viral DNA integrated into the host genome. Histogram representing copy number of viral DNA integrated into the host genome of SUP-T1, THP-1, 1321N1 and GO-G-CCM cells. The DNA region corresponding to the promoter of CXCR4 was used as an internal control to normalize the total genomic DNA. Experiments were done five times and ± SD values were determined. The significance is determined by student’s t test and p values are denoted. The bar indicates the groups compared. (TIF) [file pone.0072905.s006.tif]

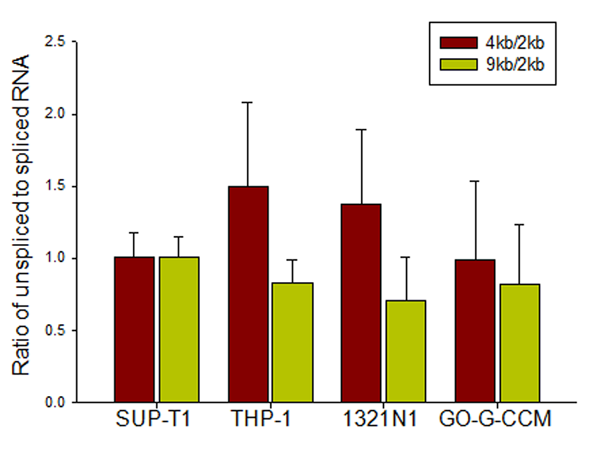

Supplement: Figure S7 — The ratio of unspliced (9 kb) or partially spliced (4 kb) to completely spliced (2 kb) RNA transcript in SUP-T1, THP-1, 1321N1 and GO-G-CCM cells. Briefly, RNA was extracted from each cell type after 6 hour post infection, qRT-PCR was performed for primers specific to 9 kb, 4 kb and 2 kb HIV-1 RNA species. Ratio of partially (4 kb) spliced or unspliced (9 kb) HIV-1 RNA to completely spliced (2 kb) RNA for respective cell lines were plotted. The ratio of unspliced (9 kb) or (4 kb) spliced HIV-1 RNA to completely spliced (2 kb) RNA in all the cell lines did not differ significantly, with all showing less than 0.5 fold differences. All experiments were done more than three times and error bars represent mean ± SD. (TIF) [file pone.0072905.s007.tif]

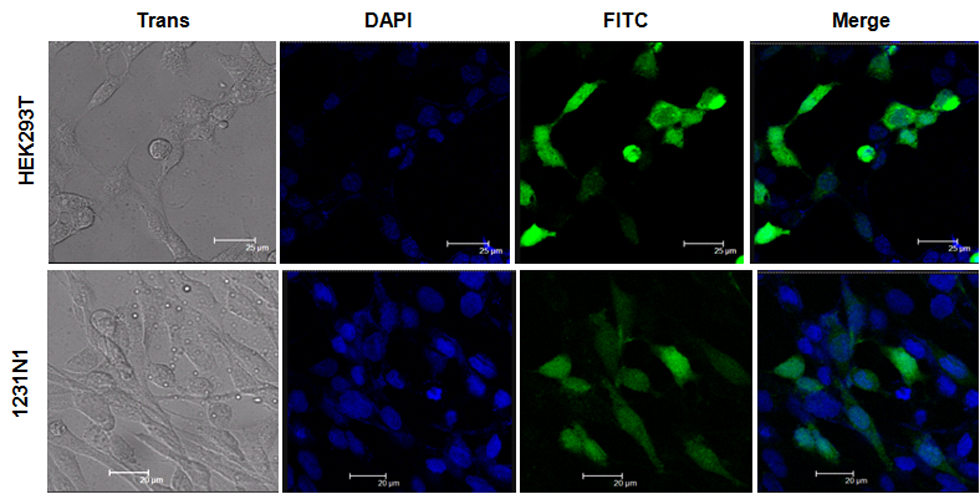

Supplement: Figure S8 — Control EGFP vector expression in astrocyte 1321N1 and HEK293T cells. Astrocyte 1321N1 and HEK293T cells were transfected with EGFP vector alone and expression was checked after 48 hours. All the experiments were done thrice and representative pictures are shown here. (TIF) [file pone.0072905.s008.tif]

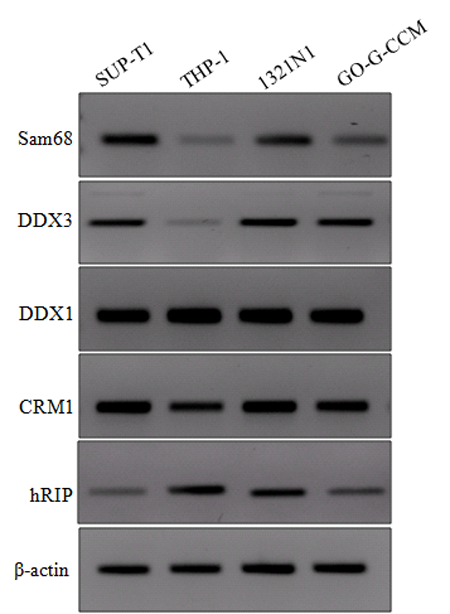

Supplement: Figure S9 — Expression profiles at the transcript levels of known Rev interacting partners. Semi-quantitative RT-PCR analyses of 6 different genes namely Sam68, DDX3, DDX1, CRM1, hRIP and β-actin in SUP-T1, THP-1, 1321N1 and GO-G-CCM. β-actin was used as a loading control. All experiments were done three times and a representative gel is shown. (TIF) [file pone.0072905.s009.tif]
