# Supplementary material for: Impact of Viral Factors on Subcellular Distribution and RNA Export Activity of HIV-1 Rev in Astrocytes 1321N1
Source: PLoS One. 2013 Sep 4;8(9):e72905. doi: 10.1371/journal.pone.0072905 (PMC3762830; doi:10.1371/journal.pone.0072905)
Supplement: Table S1 — List of primers used in the study. (DOCX) [file pone.0072905.s010.docx]

**Table S1: Primers used in the study**

| Rev-EGFP | Rev-EGFP-FP | 5’ATCATAGATCTAATGGCAGGAAGAAGC 3’ |
| --- | --- | --- |
|  | Rev-EGFP-RP | 5’CCCGTCGACAATTAATTCTCTTTAGTTC 3’ |
| SAM68 | SAM68-RT- FP | 5’ GGATATGATGATACATACGCAGAACAAAGT 3’ |
|  | SAM68 –RT-RP | 5’ CATAAGCTTCATAAGAATCTTGAACCTCC 3’ |
| DDX1 | DDX1 RT-FP | 5’ AAGGTACCAGTGGATGAATTTGATGGGAAA 3’ |
|  | DDX1 RT-RP | 5’ CAGGAAAGATGTCTGCGCCTCCTT 3’ |
| DDX3 | DDX3 RT- FP | 5’ TTCCAGCTTCAGCAGCAGCCGCGC 3’ |
|  | DDX3 RT-RP | 5’ TCAGTTACCCCACCAGTCAACCCCCTG 3’ |
| hRIP | hRIP RT- FP | 5’ GCTTTAGTGGCAGCTTTCAGCAGC 3’ |
|  | hRIP RT-RP | 5’ CATAAAAGGATTACTAGATACTCCAGCAGCTG 3’ |
| CRM1 | CRM1 FP | 5’ TTTTCAGCTTAAATCAAGATATTCCTGCTT 3’ |
|  | CRM1 RP | 5’ TAAAGATGCCAGGGACAGACATTT 3’ |
| β-Actin | Actin-RT-FP | 5’ AGCCTCGCCTTTGCCGA 3’ |
|  | Actin-RT-RP | 5’ CTGGTGCCTGGGGCG 3’ |
| CXCR4 | CXCR4-FP | 5’CACAGAGAGACGCGTTCCTAG 3’ |
|  | CXCR4-RP | 5’AGCCAACAAACTGAAGTTTCTG 3’ |
| Splicing | TAR-FP | 5’ CTGAGCCTGGGAGCTCTCTGGC 3’ |
|  | 2kb RP | 5’ CCGCAGATCGTCCCAGATAAG 3’ |
|  | 4kb RP | 5’ TCATTGCCACTGTCTTCTGCTCT 3’ |
|  | 9kb RP | 5’ TGCGAATCGTTCTAGCTCCCTGCTTGCCCATACTATATGTTT 3’ |
| Integration | Alu FP | 5’ GCCTCCCAAAGTGCT GGGATTACAG 3’ |
|  | Gag-RP | 5’ GCTCTCGCACCCATCTCTCTC C 3’ |
|  | LTR-FORWARD | 5’ GCCTCAATAAAGCTTGCCTTG A 3’ |
|  | LTR-REVERSE | 5’ TCCACACTGACTAAAAGGGTCTGA 3’ |
