# Supplementary material for: Impact of Viral Factors on Subcellular Distribution and RNA Export Activity of HIV-1 Rev in Astrocytes 1321N1
Source: PLoS One. 2013 Sep 4;8(9):e72905. doi: 10.1371/journal.pone.0072905 (PMC3762830; doi:10.1371/journal.pone.0072905)
Supplement: Materials and Methods S1 — (DOCX) [file pone.0072905.s011.docx]

**MATERIALS AND METHODS**

**Expression levels of 2, 4 and 9kb HIV-1 mRNA**

RNA was isolated from all the cell lines by Trizol method after 6 hours post infection and cDNA was prepared by using random hexamer and superscript III enzyme (Invitrogen, USA). Equal amount of cDNA was used for qRT-PCR to amplify 2, 4 and 9 kb products. For all the mRNA splice variants, forward primer TAR-FP and respective 2kb-RP, 4kb-RP and 9kb-RP (Table S1) were used to amplify respective RNA species as described earlier [1]. No false amplification was detected in uninfected control. Keeping 2kb RNA as a control, the abundance of 4 kb and 9 kb were analyzed by qRT-PCR method with formula 2^-ΔΔCT^.

**RT-PCR analysis of SAM68, DDX1, DDX3, hRIP and CRM1**

RNA was isolated from all the cell lines by Trizol method and cDNA was prepared by using oligo dT and superscript III enzyme (Invitrogen, USA). Equal amount of cDNA was used to amplify *sam68, ddx1, ddx3, hrip* and *crm1* genes using specific primers listed in table S1. All the primers are designed at the splice junction and negative controls were checked to rule out DNA contamination. Gene for β-actin was used as a loading control. Samples without reverse transcriptase were taken as negative controls.

1. Chatel-Chaix L, Clement JF, Martel C, Beriault V, Gatignol A, et al. (2004) Identification of Staufen in the human immunodeficiency virus type 1 Gag ribonucleoprotein complex and a role in generating infectious viral particles. Mol Cell Biol 24: 2637-2648.
